# Fibro-Stenosing Crohn’s Disease: What Is New and What Is Next?

**DOI:** 10.3390/jcm12093052

**Published:** 2023-04-22

**Authors:** Virginia Solitano, Arianna Dal Buono, Roberto Gabbiadini, Marek Wozny, Alessandro Repici, Antonino Spinelli, Stefania Vetrano, Alessandro Armuzzi

**Affiliations:** 1 Department of Biomedical Sciences, Humanitas University, Pieve Emanuele, 20090 Milan, Italy; virginia.solitano@humanitas.it (V.S.); marek.wozny@hunimed.eu (M.W.); alessandro.repici@hunimed.eu (A.R.);; 2 Division of Gastroenterology, Department of Medicine, Western University, London, ON N6A 4V2, Canada; 3 IBD Center, IRCCS Humanitas Research Hospital, Rozzano, 20089 Milan, Italy; arianna.dalbuono@humanitas.it (A.D.B.); roberto.gabbiadini@humanitas.it (R.G.); 4 Department of Endoscopy, IRCCS Humanitas Research Hospital, Rozzano, 20089 Milan, Italy; 5 Division of Colon and Rectal Surgery, IRCCS Humanitas Research Hospital, Rozzano, 20089 Milan, Italy

**Keywords:** fibrosis, stricture, IBD, cross-sectional imaging, balloon, stricturoplasty

## Abstract

Fibro-stenosing Crohn’s disease (CD) is a common disease presentation that leads to impaired quality of life and often requires endoscopic treatments or surgery. From a pathobiology perspective, the conventional view that intestinal fibro-stenosis is an irreversible condition has been disproved. Currently, there are no existing imaging techniques that can accurately quantify the amount of fibrosis within a stricture, and managing patients is challenging, requiring a multidisciplinary team. Novel therapies targeting different molecular components of the fibrotic pathways are increasing regarding other diseases outside the gut. However, a large gap between clinical need and the lack of anti-fibrotic agents in CD remains. This paper reviews the current state of pathobiology behind fibro-stenosing CD, provides an updated diagnostic and therapeutic approach, and finally, focuses on clinical trial endpoints and possible targets of anti-fibrotic therapies.

## 1. Introduction

Fibrosis represents a major challenge in the management of Crohn’s disease (CD) [1]. Around 50% of CD patients will develop fibrotic strictures or penetrating lesions, and up to 75% will eventually need surgery [2]. Nevertheless, it is common for patients to experience post-operative recurrence of fibrosis, especially at the ileocolonic anastomotic site, which might lead to the onset of re-stricturing disease and necessitate further surgeries [3].

The mechanism underlying the development and progression of fibrosis in inflammatory bowel disease (IBD) is still unclear, but growing evidence suggests that chronic intestinal inflammation, causing repetitive unphysiological healing of injured tissue, represents the main driver [4]. However, fibrotic changes persist once inflammatory stimuli are eliminated, and suppression of inflammation does not necessarily have an effect on intestinal fibrosis [4]. Multi-modal single-cell approaches are revealing a deeper understanding of the role of distinct cell populations [5].

Furthermore, a lack of accepted clinical trial endpoints and treatment targets for fibro-stenosis, as well as the extreme heterogeneity in disease definitions, diagnostic techniques, and study design, led an international study group to develop a framework for trial design and endpoints to help researchers and regulators in the development of new drugs [6].

The aim of this review is to present the state of the field in relation to the most recently identified pathogenic mechanisms of fibro-stenosing CD, focusing on current management strategies, possible targets of anti-fibrotic therapies, and new perspectives. 

## 2. Molecular Pathways of Fibrosis in Crohn’s Disease

**Figure 1:**
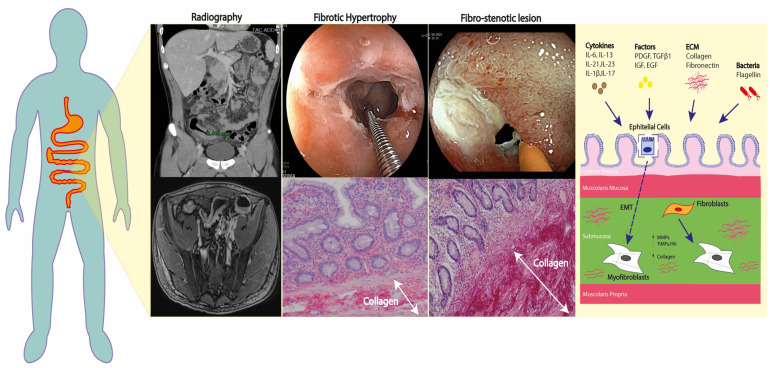
Overview of the main pathophysiological mechanisms behind fibro-stenosing CD.

Fibro-stenosing lesions in CD patients display specific histological features, such as thickening of the muscularis mucosae and muscularis propria that causes hardening of the intestinal wall and luminal narrowing [7,8]. Hypertrophy of the muscularis propria and smooth muscle hyperplasia of the submucosa are highly predominant in fibro-stenosing lesions; while the fibrosis itself, defined as a deposit of collagenous fibers, contributes to a lesser extent [7,8]. Despite remaining only partly understood, fibrogenesis in IBD depends on two parallel and simultaneous processes: the expansion of the smooth muscle layers and the growth of the extra-cellular matrix (ECM) in all layers [9]. The process of stricture formation is long and continuous, and it only becomes evident in late stages when the lesions produce symptoms of bowel obstruction (Figure 1). The gradual change in the ECM, and the cellular composition and distribution within the intestinal wall layers, implicate both inflammatory and non-inflammatory pathways [10,11]. Activated fibroblasts, defined as myofibroblasts, and smooth muscle cells, initiate the cascade of gastrointestinal fibrogenesis through the secretion of growth factors and ECM proteins [12,13]. The triggering stimuli that are able to activate myofibroblasts include chronic exposure to autocrine and paracrine soluble inflammatory factors (i.e., interleukins [ILs], platelet-derived growth factor subunit A [PDGFA], PDGFB, transforming growth factor beta 1 [TGFβ1], insulin-like growth factor 1 [IGF-I], epidermal growth factor [EGF]) [14], as well as some components of the ECM itself (i.e., fibronectin) [15,16], the Wnt-β-catenin signaling pathway, and pathogen-associated and damage-associated molecular patterns (PAMs, DAMs) [12,13]. Membrane glycolipids and/or soluble molecules deriving from microorganisms, together with free nucleic acids (i.e., DNA, RNA), fragments of ECM elements, and ATP, interact with toll-like receptors (TLRs); mainly TLR-4, thus affecting the myofibroblast activity [14,17]. Concerning inflammation, the induced altered pattern of DNA methylation can modify gene expression, thus mediating intestinal fibrosis, as demonstrated in CD-associated chronic inflammation [18]. The lymphocytic profile that is primarily involved appears to be Th17+, as these cells promote intestinal fibrosis through IL-6, IL-21, IL-23, IL-1b, and IL-17, signaling in both experimental mice models and in human patients with CD [19].

Additionally, this process self-perpetuates through intense epithelial-to-mesenchymal transition: it has been demonstrated that endothelial and epithelial cells can change phenotype and contribute to the ECM deposition [20]. Cells that have undergone epithelial-to-mesenchymal transition lose their epithelial imprinting, morphology, and function, acquiring spindle-shape morphology and fibro-blast markers such as alpha-smooth muscle actin (αSMA). This is demonstrated by the co-expression of epithelial markers such as E-cadherin and mesenchymal markers such αSMA or vimentin, both in in vivo animal models [21] and in fibrotic areas of intestinal tissue specimens and biopsies of patients with IBD [22,23]. In fibro-stenosing lesions, the signaling between mesenchymal cells and the surrounding immune cells becomes bidirectional with paracrine secretion of multiple factors.

A growing body of evidence suggests the involvement of the gut microbiota in the pathogenetic mechanism in fibrogenesis. In addition to indirectly activating fibrotic processes by causing inflammatory responses, the gut microbiota can directly stimulate pro-fibrotic processes in myofibroblasts [8,9,10]. When deprived of microflora, animal models do not develop intestinal fibrosis, and specific actions of bacterial components such as flagellin have been recognized as able to activate intestinal mesenchymal cells [24,25].

Ultimately, during intestinal fibrogenesis the physiological degradation of ECM is dysregulated via various tissue inhibitors of metalloproteinases (MMPs), including the tissue inhibitors of metalloproteinases (TIMP1) and the plasminogen activator inhibitor-1 (PAI-1) produced by both immune cells and mesenchymal cells [26,27]. Accordingly, increased levels of TIMPs and PAI-1 have been measured in the muscle layers of ileal strictures in CD [28].

## 3. Assessment of Fibro-Stenotic Disease in CD

Despite substantial heterogeneity in defining fibro-stenotic strictures, the fundamental elements for radiological definition are luminal narrowing, bowel wall thickness, and the presence of pre-stenotic dilation [29,30,31]. The CrOhN’s disease anti-fibrotic STRICTure therapies (CONSTRICT) group provided accurate criteria for each item, defining the luminal narrowing as >50% compared to an adjacent healthy bowel tract, the bowel wall thickening as a 25% increase in wall thickness compared to the adjacent healthy bowel tract, and the pre-stenotic dilation as at least ≥3 cm in diameter [6]. The ability to distinguish between fibro-stenotic-predominant from inflammatory-predominant strictures using the different available imaging techniques has been extensively investigated, but currently no imaging technique is able to accurately quantify the amount of fibrosis in a stricture.

### 3.1. Bowel Ultrasound and Ultrasound Elastography

Regarding bowel ultrasound (BUS), several findings have been associated with the presence of fibrosis, such as bowel wall thickness with lack of vascularity and/or contrast enhancement, and possibly increased parameters at elastography [29,30]. BUS is an operator-dependent technique and the diagnostic accuracy requires an experienced sonographer with specific expertise in small bowel investigation. Currently, available data on the accuracy of BUS in discriminating fibrosis within bowel stricture are scarce: in a recent meta-analysis of 14 studies, BUS was considered inaccurate in differentiating fibrotic from inflammatory stenosis [32]. This meta-analysis confirmed that fibro-stenosing lesions in CD present a thicker bowel wall and a lower enhancement [32]. Maconi et al. reported a sensitivity of 100% and a specificity of 63–75% of the BUS echo pattern in identifying a moderate to severe degree of fibrosis in the submucosa and the muscularis mucosae [33,34]. Contrastingly, studies of the small intestine contrast ultrasonography (SICUS) estimated a sensitivity of 88–97% and a specificity of 88–100% of this technique, using histopathological evaluation of surgical resections as reference standard [34].

In a recent prospective study that investigated BUS and elastography in 20 CD patients, despite a rather low inter-reader agreement (k = 0.38 for all techniques combined), the authors found that the combination of conventional B-mode ultrasound, contrast-enhanced ultrasound, and strain elastography increased the diagnostic accuracy and diagnostic confidence in detecting mural fibrosis, as compared to the single techniques alone [35].

Finally, the accuracy of ultrasound elastography largely varies among different studies (35–90%). The available data do not allow a precise estimate of this modality’s ability to recognize mural fibrosis, mainly due to the high heterogeneity of the elastography modalities (strain- vs. shear-wave elastography) and the histological assessment of fibrosis [36].

### 3.2. Magnetic Resonance (MR) and MR-Enterography (MRE)

MRE is a radiation-free, accurate, and widely available imaging technique for stricture diagnosis and differentiation. The estimated sensitivity and specificity of MRE for stricture detection are >90% [29,37], which makes it the recommended test for assessing and monitoring CD fibro-stenosing lesions [6,29,30]. Among the different modalities, contrast-enhanced MR (CE-MR), diffusion-weighted MRE (DW-MR), delayed gadolinium enhancement MR, and magnetization transfer MR (MT-MR) are utilized.

Despite the lack of full validation, MR parameters have been able to predict fibrosis. Wall thickness on T2-weighted and T1-weighted sequences, as well as T2-weighted mural hyperintensity, significantly correlated with fibrosis (*p* < 0.05) in a retrospective series of consecutive CD patients [38]. Further studies reported that mural thickness, T1 ratio, maximal enhancement, and apparent diffusion coefficient (ADC) values significantly reflected the grade of fibrosis severity (*p* < 0.05) [39]. Notably, MRE acquisitions performed some minutes after contrast administration (i.e., when exhibiting the so-called delayed enhancement) accurately distinguished between different grades of fibrosis severity in surgical specimens, with a sensitivity of 94% and a specificity of 89%, respectively [40]. Others have shown that diffusion-weighted imaging (DWI) values (an enhanced diffusion signal and a corresponding low ADC) correlate inversely with fibrosis (*p* < 0.05) [41], and the combination of the MR Index of Activity (MaRIA) score and the ADC reached a specificity of 93% [42].

A prospective study evaluating MT-MR, which produces image contrast based on the concentration of large macromolecules (i.e., collagen) in tissues, found that normalized MT ratios were significantly associated with fibrosis (r = 0.77, *p <* 0.001) [43]. When compared to the ADC determined by DW-MR, the MT ratio showed a superior accuracy (higher AUC), being able to distinguish between non-fibrotic and differently graded wall fibrosis (*p* = 0.001) [43].

More recently, a type I collagen-targeted MR imaging probe was used to stage intestinal fibrosis CD in a mouse model; its effectiveness was compared to that of the MR aging contrast medium gadopentetatedimeglumine (Gd-DTPA) [44]. The probe had a better enhanced effect and an increased impact compared to Gd-DTPA. As such, the imaging probe is attractive for assessment of the development and tracking of the therapeutic response in intestinal fibrosis in CD patients. 

### 3.3. Computed Tomography (CT)

As with MR, the accuracy of CT techniques including CT enterography (CTE) approaches 90–100% in diagnosing strictures in CD patients [6,30,31]. However, scarce data are available on its ability to characterize CD-associated fibro-stenosing lesions [45,46,47]. A cohort study of 54 CD patients found that the severity of the stricture detected by CT significantly correlated with histologically confirmed fibrosis in surgical specimens (*p* = 0.007), and the adopted CT score displayed a sensitivity of 79% in detecting fibrosis [45]. CT and CTE parameters, especially mesenteric hypervascularity, mesenteric fat stranding, and mucosal hyperenhancement, are extremely accurate in predicting bowel inflammation [47], while the prediction of tissue fibrosis appears limited through this technique [47].

## 4. Management of Fibro-Stenosing CD

**Figure 2:**
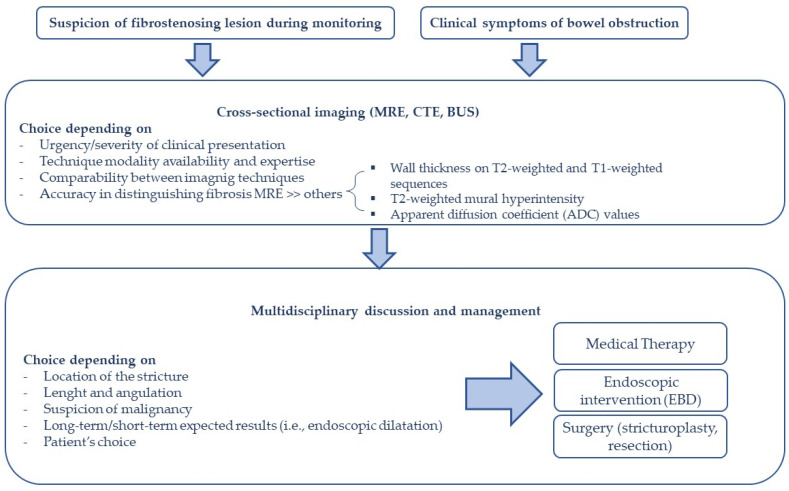
Proposed algorithm for fibro-stenosing CD.

Managing the treatment of individuals with fibro-stenosing CD is challenging and requires a multidisciplinary team comprising a gastroenterologist, a colorectal surgeon, and a radiologist to evaluate the appropriate strategy (Figure 2) [48]. The optimal therapeutic approach changes according to the features of the stenosis (i.e., location, length, angulation), the presence of any associated complications (i.e., fistula, abscess, dysplasia), and the patient’s preference [49,50]. 

### 4.1. Medical Therapy

CD patients with fibro-stenosing lesions are currently treated with the best available medical therapies, given that specific anti-fibrotic therapies are only available within clinical trials. The evidence for the effectiveness of medical treatments in fibrosis-predominant lesions remains very limited. The available data on corticosteroids have shown their efficacy in reducing obstructive symptoms in almost 100% of patients; however, this method has high recurrence rates (around 50%) and often requires surgery [51]. It is worth noting, in experimental mice models of CD, a one-week treatment with prednisolone administered via enemas resulted in anti-fibrotic effects at histological assessment [52]. Such anti-fibrotic effects of steroids were mediated by TRPA1 channel activation concomitant with an anti-TGF-β action [52].

In the past, the efficacy of anti-tumor necrosis factor (TNF) agents in fibro-stenosing CD has produced conflicting results. Previous studies have reported high clinical response after infliximab in CD patients with strictures [53,54]. However, concerns were raised about anti-TNFs promotion of fibrosis through extremely rapid tissue healing and scarring [11,54]. Subsequently, robust evidence confuted these concerns: neither progression in strictures’ severity nor new occurrence of strictures were repeatedly observed [55,56,57], and possible mechanisms mediated by anti-TNFs able to prevent fibrosis have been suggested [58]. In the multi-center, prospective CREOLE study, adalimumab treatment was successful in approximately 60% of CD patients with strictures and obstructive symptoms after 6 months of treatment, and 30% of the patients continued to be in clinical remission after a median follow-up time of 4 years (still on adalimumab, without any endoscopic or surgical treatment) [57]. Over the 4-year study period, slightly more than 50% of the included patients remained free of surgery after commencing adalimumab [57]. Finally, the STRIDENT trial, which included CD patients with evidence of stricturing disease and associated chronic or subacute intestinal obstruction, showed high rates of clinical and radiological response (assessed on BUS and MRE) in both adalimumab arms (intensive vs. standard treatment) [59]. 

Still, a certain heterogeneity in the characteristics of the lesions analyzed in the above-mentioned studies might bias clear conclusions on the effectiveness of both steroids and anti-TNFs in fibrosis-predominant strictures.

### 4.2. Endoscopic Therapy 

No medical therapy that prevents or reverses fibrosis is currently available in clinical practice and these patients are generally managed with endoscopic balloon dilation (EBD) or surgery [60,61].

EBD is a cost-effective, minimally invasive procedure for handling symptomatic short CD strictures [60,61,62,63]. The procedure ameliorates obstructive symptoms and can be repeated if the stricture re-occurs, improving the long-term prognosis of subjects with CD [64]. Furthermore, EBD may also have an important role as a bridge-to-surgery therapy, allowing the improvement of the patient’s nutritional status [49]. EBD should be avoided in stenosis associated with problems such as fistula, abscess, or underlying dysplasia; however, it can be used in naive or anastomotic, straight (non-angulated) strictures that are less than 5 cm in length [50,63,65]. Generally, the most used device for EBD is a through the scope (TTS) radial expanding balloon dilator which pneumatically dilates the stricture [63]. Graded dilation up to the largest size (18–20 mm) in a stepwise mode is recommended to obtain maximum efficacy [65]. Indeed, in the study by Reutmann et al., patients who failed to achieve a maximum dilation of 14 mm or more had an increased risk of surgery (hazard ratio [HR] 2.88; 95% CI 1.10–7.53), while those reaching a dilation of 16–18 mm displayed a longer interval between EBDs (mean 240 ± 136.7 days vs. 456 ± 357.3 days) [66]. Overall, EBD exhibited optimal rates of immediate technical success, typically defined as the possibility to pass the stenosis with the endoscope after the procedure (technical success of 89.1% with clinical efficacy of 80.8%) [67]. Regarding long-term outcomes, Bettenworth et al. observed that re-dilation for ileal and anastomotic strictures was needed in 36.5%, 51.8%, and 73.5% of cases at 6, 12, and 24 months, respectively; while 17.5%, 30.1%, and 42.9% of patients required surgery at 6, 12, and 24 months, respectively [67]. The length of the stricture was a predictor of surgery-free interval (≤5 cm stricture was associated with a longer surgery-free survival time, HR 2.5; 95% CI 1.4–4.4) [67]. 

Conversely, although EBD can also be attempted in colonic stenosis, surgical intervention should be considered in these cases due to the increased risk of malignancy and because stricture biopsy may not rule out the presence of dysplasia [50,68]. 

EBD has low rates of adverse events (i.e., perforation, bleeding, abscess, or dilation-related need for surgery) which are estimated to be 2.8–4% [67,69]. Unlike long-term outcomes, stricture length does not seem to be associated with the risk of bleeding or perforation. However, the use of systemic steroids may be positively associated with post-EBD perforation while fibrotic stricture has a potential protective role [70]. 

Other endoscopic techniques have been proposed for CD-related strictures. Endoscopic stricturotomy is an emergent technique in which a needle knife or an insulated-tip knife is used for radial, circumferential, or horizontal incisions of a stenotic bowel tract to progressively widen the bowel lumen [65,71,72]. Although data are limited, this technique exhibits high immediate technical success rates [73], with excellent rates of reported clinical and endoscopic improvement [74]. A lower risk of perforation has been reported (0% vs. 2.4%, albeit p not significant), but the higher risk of bleeding compared to EBD (14% vs. 0%, *p* < 0.001) has raised some concerns [74]. Thus, the role of endoscopic stricturotomy in the treatment of CD strictures has yet to be defined and more studies are needed to understand the real efficacy and safety of this technique. 

Similarly, some reports have described the use of stents as an alternative treatment for CD-related strictures with high rates of improvement or resolution of symptoms [75,76,77].

In a recent multi-center randomized trial, individuals with CD-related strictures, which were treated with EBD, more often avoided a new therapeutic intervention at one year compared to those treated with fully covered self-expandable metal stents (FCSEMS) (80% vs. 51% respectively; OR 3.9; 95% CI 1.4–10.6) [78]. It is noteworthy that success rates were around 65% for both procedures when the length of the stricture was >3 cm, hence FCSEMS may have a role in the treatment of longer strictures [78]. More research is required to determine SEMSs proper place in the endoscopic management of CD strictures; for now, EBD is still the preferred technique.

### 4.3. Surgical Therapy

Symptomatic CD strictures that are not manageable with EBD should be referred for surgery, namely surgical resection or strictureplasty [50,71,79]. Early surgical resection, shortly after diagnosis, should be the preferred strategy for localized ileocecal fibro-stenosing CD [48]. Indeed, many studies showed that early surgery may prolong clinical remission and reduce the need for subsequent biologic therapy, or the risk of further surgery compared to late surgery (surgery performed during the disease) [80,81,82]. 

Interestingly, Li et al. observed that patients with ileocolonic anastomotic stricture, which underwent salvage surgery after one or more EBDs, were associated with increased rates of stoma formation (*p* = 0.030) and surgical-site infection (*p* = 0.025), compared with individuals who underwent immediate surgery [83]. Furthermore, early surgery may also be considered the best therapeutic option over EBD for primary CD ileocolic strictures. In a retrospective study conducted by Lan et al., surgical resection of primary CD ileocolic strictures was associated with a reduced need for secondary surgery and a longer surgery-free time interval compared to individuals managed with EBD (11.1 ± 0.6 vs. 5.4 ± 0.6 years; *p* < 0.001) [84].

Strictureplasty is a validated and safe surgical choice for the management of CD strictures and is an alternative to bowel resection [85,86]. This surgical technique is recommended for multiple fibrotic strictures or previous extensive resections, thus protecting against small bowel loss [61]. However, strictureplasty is not always technically feasible, and it is not recommended in the colon, in cases of penetrating or actively diseased terminal ileum. 

The Heineke-Mikulicz and Finney strictureplasties are the most common techniques used for short (<10 cm) and medium length (10–20 cm) strictures of the small bowel, respectively; while the “non-conventional” Michelassi side-to-side isoperistaltic strictureplasty, with or without mid-stricture resection, is suggested for long strictured tracts (>20 cm) and is less commonly performed [50,87]. Interestingly, recurrence rates are low and the quality-of-life post-strictureplasty is comparable with surgical resection [88]. In a meta-analysis conducted by Yamamoto et al., (1112 individuals and 3259 stricturoplasties included) the recurrence rate at 5 years was 28% [89]. Adverse events of this surgical intervention have been described in 4% to 18% of individuals and comprise surgical site infection, obstruction, stricture-site hemorrhage, sepsis, perforation, and early reoperation [79,86,89,90,91,92]. It is important to note that low albumin, weight loss, older age, preoperative steroid therapy, and the presence of abscess or fistula at the moment of strictureplasty increase the risk of intra-abdominal septic adverse events [79]. Therefore, optimization of nutritional status is a crucial measure for the best management of these patients [50].

## 5. The Role of Stenosis Therapy and Anti-Fibrotic Research (STAR) Consortium 

Despite the important developments in the field of fibrosis, the definitions for stricturing IBD on endoscopy and cross-sectional imaging are extremely heterogeneous in the current literature. Another significant barrier is the absence of established clinical trial goals, precise biomarkers, or imaging techniques that may reliably quantify fibrosis in a stricture [93]. 

The global consortium Stenosis Therapy and Anti-Fibrotic Therapy (STAR) created clear definitions for a stricture and is building endpoints to test anti-fibrotic agents in CD [94]. In 2018, using a modified RAND/UCLA appropriateness technique, this group carried out an international consensus process to standardize CD stricture definitions, inclusion criteria, and endpoints for use in everyday clinical practice [6]. Based on the items considered appropriate, the STAR group built a prototypic clinical trial design. 

First, candidates must have a single, naive, or anastomotic ileal stricture that is reachable by endoscopy and confirmed by cross-sectional imaging (CT or MR enterography). The three features—localized luminal narrowing (luminal diameter decrease of at least 50%), gut wall thickening (wall thickness increased by at least 25%), and pre-stenotic dilation—should be present in all strictures on cross-sectional imaging, as was previously discussed. According to STAR, an endoscopic stricture is the inability to pass an adult colonoscope without previous dilatation and appropriate pressure. The remission of symptoms along with a concurrent decrease in luminal narrowing, pre-stenotic dilatation, wall thickening, and stricture length should be considered the success of anti-fibrotic therapy. Lastly, the optimal time to evaluate treatment success was 24 weeks after remission. 

The purpose of a later RAND/UCLA exercise was to assess the suitability of histopathology scoring systems and items created based on panel opinion, thereby standardizing CD histopathology criteria to further establish a CD stricture histopathology score [95]. None of the scoring systems used to assess CD strictures were considered appropriate for clinical trials. A stricture should be defined as an increased thickness of all layers of the bowel wall, fibrosis of the submucosa and bowel wall, and muscularization of the submucosa. Attention should also be given to the distinction between active inflammation (neutrophils, erosion, and ulcers) and chronic mucosal injury (architectural changes). Core projects of the STAR consortium include the development of a patient-reported outcome tool, a stricture radiology index, and a stricture histopathology index.

## 6. Therapeutic Potential: What Is Next 

The paradigm of CD-related inflammation that leads to irreversible fibro-stenotic stricture formation, obstruction, and surgery is being revisited. Other organ systems, including the skin, liver, and kidney, have shown reversible fibrosis, and a better understanding of the mechanisms that cause fibrosis has enabled the development of an expanding pipeline of anti-fibrotic medications that specifically target fibrogenesis-related molecular pathways [96]. Corroborating this, Zhao et al. recently described how IL-6/IL-21–Stat3 signaling drives Areg production in Th17 cells, revealing a pro-fibrotic mechanism that, contrary to current approaches, acts independently of intestinal inflammation [19]. From a clinical perspective, these findings reinforce the hypothesis that Th17 cells promote intestinal fibrosis via inflammation-independent Areg expression. 

Anti-fibrotic agents have been developed and have shown promise in other organs [97]. Pirfenidone and nintedanib have been approved for the treatment of idiopathic pulmonary fibrosis and represent potential future options in fibro-stenosing CD. These two drugs provide great examples of a multi-target approach to several cytokines and growth factors, acting on inflammation and fibrosis together. The former inhibits TGF-β and TNFα, whereas the latter is thought to inhibit PDGF, fibroblast growth factor (FGF), and vascular endothelial growth factor (VEGF) receptor signaling [98,99]. Interestingly, AGMB-129, an oral small molecule GI-restricted inhibitor of TGFβ type I receptor kinase (ALK5), showed a favorable safety profile in a phase 1 study, and a phase 2a trial is planned in patients with fibro-stenosing CD.

The myofibroblast is another final effector cell that induces fibrosis. Possibilities for anti-fibrotic therapy increase when these cells are specifically targeted, or when myofibroblast precursor cell activation is prevented. It would be beneficial to employ methods that block their accumulation, for instance, by preventing their differentiation into myofibroblasts. In view of this, it was found that the Rho-associated protein kinase inhibitor AMA0825 reversed intestinal fibrosis in mice and decreased the release of pro-fibrotic markers in CD biopsies, such as matrix MMPs, collagen, and IL6 [100]. 

In tissues from patients with fibro-stenosing CD, significantly higher levels of IL-36A were found [101]. Inhibition of the IL-36 receptor via an antibody to the IL-36 receptor significantly reduced established fibrosis in mice with chronic intestinal inflammation [102]. Along these lines, spesolimab, a monoclonal antibody against IL-36 receptor, showed significant clinical improvement in patients with generalized pustular psoriasis [103], and a phase 2 randomized controlled trial is currently ongoing in patients with stricturing CD (NCT05013385).

Finally, noncoding RNAs, including microRNAs long noncoding, RNAs, and circular RNAs have been proven to play a role in fibrotic diseases of multiple organs such as kidney, liver, and heart [104]. Research has shown that noncoding RNAs influence the cytokines or chemokines that are relevant to the inflammation, the activation of immune cells, such as lymphocytes Th1 and Th17, and their differentiation [105,106]. Additionally, noncoding RNAs regulate the tight junctions of the gut mucosa, the mucus barrier, and immunological homeostasis to affect the function of the intestinal epithelial barrier [107]. Noncoding RNAs may someday become potential diagnostic and therapeutic targets as the pathophysiology of noncoding RNA-modulated intestinal fibrosis is gradually uncovered.

## 7. Conclusions

The management of fibro-stenosing CD should be decided in consultation with a multidisciplinary team. To lessen the pro-inflammatory mediators of fibrosis, luminal inflammation must be controlled with anti-inflammatory therapies, including steroids and biologic agents. Endoscopic and surgical interventions may be required in those patients with disease complications or those who are not responsive to medical therapy alone.

Nevertheless, given that fibrosis can advance once established regardless of the suppression of inflammation, anti-fibrotic medicines are now focusing on targeting mechanisms that are independent of inflammation. Drugs evaluated for fibrosis in other organs may also be applicable to intestinal fibrosis, as the pathophysiological mechanisms involved in transmural intestinal fibrosis may be comparable. Despite extensive study, there are currently no available medications for intestinal fibrosis; instead, pharmacological treatments are only available for fibrotic disorders such as idiopathic pulmonary fibrosis. However, a significant amount of work has been completed to develop a prototype clinical trial for structuring CD and to standardize stricture definitions. Remodeling drugs that can reverse the phenotype are eagerly awaited for use in combination with already available anti-inflammatory agents.

## Data Availability

Not applicable.
